# Cognitive behavioral therapy for insomnia in euthymic bipolar disorder: study protocol for a randomized controlled trial

**DOI:** 10.1186/1745-6215-15-24

**Published:** 2014-01-16

**Authors:** Mette Kvisten Steinan, Karoline Krane-Gartiser, Knut Langsrud, Trond Sand, Håvard Kallestad, Gunnar Morken

**Affiliations:** 1 Department of Neuroscience, Norwegian University of Science and Technology (NTNU), Trondheim, Norway; 2 Østmarka Department of Psychiatry, St. Olav’s University Hospital, Trondheim, Norway

**Keywords:** Bipolar disorder type I and II, CBT-I, Cognitive behavioral therapy, Euthymic, Insomnia, Randomized controlled trial

## Abstract

**Background:**

Patients with bipolar disorder experience sleep disturbance, even in euthymic phases. Changes in sleep pattern are frequent signs of a new episode of (hypo)mania or depression. Cognitive behavioral therapy for insomnia (CBT-I) is an effective treatment for primary insomnia, but there are no published results on the effects of CBT-I in patients with bipolar disorder. In this randomized controlled trial, we wish to compare CBT-I and treatment as usual with treatment as usual alone to determine its effect in improving quality of sleep, stabilizing minor mood variations and preventing new mood episodes in euthymic patients with bipolar disorder and comorbid insomnia.

**Methods:**

Patients with euthymic bipolar I or II disorder and insomnia, as verified by the Structured Clinical Interview for DSM Disorders (SCID-1) assessment, will be included. The patients enter a three-week run-in phase in which they complete a sleep diary and a mood diary, are monitored for seven consecutive days with an actigraph and on two of these nights with polysomnography in addition before randomization to an eight-week treatment trial. Treatment as usual consists of pharmacological and supportive psychosocial treatment. In this trial, CBT-I will consist of sleep restriction, psychoeducation about sleep, stabilization of the circadian rhythm, and challenging and correcting sleep state misperception, in three to eight sessions.

**Discussion:**

This trial could document a new treatment for insomnia in bipolar disorder with possible effects on sleep and on stability of mood. In addition, more precise information can be obtained about the character of sleep disturbance in bipolar disorder.

**Trial registration:**

ClinicalTrials.gov: NCT01704352.

## Background

Bipolar disorder (previously known as manic-depressive illness) has been ranked seventh among the worldwide burden of nonfatal diseases [1], and is among the most costly disorders to affect human beings [2]. In many patients, bipolar disorder has a chronic and remitting nature characterized by periods of depression and periods of hypomania or mania, and is associated with disability in social and occupational function.

Between 50% and 100% of patients with bipolar disorder have been found to suffer from sleep disturbance, even in euthymic phases [3]. Sleep could be a key factor in bipolar disorder, as sleep has been shown to be vital for emotional regulation, and a change in sleep is one of the most frequent first signs of a new episode of (hypo)mania or depression [3].

The main interventions for sleep disturbances in bipolar disorder are regulation of the pharmacological treatment and focusing on circadian regularity in psychosocial treatments [3]. Psychosocial treatments for bipolar disorder are based on around 20 sessions and consist of a package of lifestyle changes, such as regulation of activity and sleep, reduced use of alcohol and substances, monitoring signs of new episodes, support, and the formation of a plan for early intervention. Monitoring symptoms and early intervention for signs of mania are the only elements in the treatment packages that have been tested and found effective as a stand-alone intervention [4,5].

Insomnia is defined as difficulty in initiating or maintaining sleep, or nonrestorative sleep. The disturbed sleep or associated daytime fatigue causes distress or impairment [6]. Insomnia may be a primary disorder or it may be comorbid with other disorders. Insomnia affects 10% to 12% of the general population [7-9]. Despite focusing on sleep and circadian regularity in existing treatments of bipolar disorder, there may be room for improvement in the treatment of sleep [3]. To our knowledge, studies describing more focused interventions using well-documented techniques in treating insomnia in bipolar patients have not been published.

Several trials have demonstrated that cognitive behavioral therapy for insomnia (CBT-I) is an effective treatment for primary insomnia [10]. It can be superior to pharmacological treatment in both short-term and long-term perspectives [11]. Although evidence of the effectiveness of CBT-I has existed for more than 20 years, it has not been introduced to patients who also suffer from bipolar disorder [12]. Between-group effect sizes for CBT-I are usually in the range of 1.0 to 1.6, as measured with the Insomnia Severity Index (ISI) [12,13].

Although it is well documented that sleep is disturbed in bipolar disorder, there is less knowledge about how sleep is disturbed [3]. There is evidence to suggest that sleep in bipolar disorder is characterized by circadian rhythm disturbance, as in patients with delayed sleep phase syndrome, as well as by maladaptive cognitive and behavioral strategies, as in otherwise healthy individuals with insomnia [3]. Descriptive studies of frequency and type of sleep disturbance in euthymic patients with bipolar disorder are needed to improve treatment. Data from this trial will be used in a micro- and macroscopic description of the character of sleep disturbance in patients with euthymic bipolar disorder and comorbid sleep disturbance.

Sleep diaries, actigraphy, and, more seldom, polysomnography have traditionally been used to measure the effects of sleep interventions. Polysomnography is considered the best objective monitoring system and the only way to assess the constitution of sleep stages, including changes in slow wave sleep and rapid eye movement sleep, whereas subjective descriptions of sleep quality are better predictors of functional impairment and emotional distress [11].

The aims of this randomized controlled trial are to compare CBT-I and treatment as usual (TAU) with treatment as usual alone in improving quality of sleep, stabilizing minor mood variations, and preventing new mood episodes in euthymic patients with bipolar disorder and comorbid insomnia. In addition, the study will compare the use of sleep diaries, actigraphy, and polysomnography, in order to possibly simplify registrations of sleep for clinical use.

## Methods

### Study design

The study is a randomized controlled trial aimed to study the efficacy of CBT-I in euthymic bipolar disorder patients with comorbid insomnia compared with TAU. Patients will be recruited from the Department of Psychiatry, St. Olav’s University Hospital, Trondheim, Norway. They must fulfill criteria for SCID-1 (Structured Clinical Interview for DSM Disorders) verified bipolar I or II disorder and be euthymic, as defined by a score on the Montgomery Åsberg Depression Rating Scale (MADRS) not higher than 13 or by a score on the Young Mania Rating Scale (YMRS) not higher than seven. In addition, patients must fulfill the DSM-IV diagnosis criteria of ‘insomnia related to another mental disorder’ as assessed by the Insomnia Interview Schedule (IIS) [14]. Patients will not be accepted if they meet any of the following exclusion criteria: they are in an affective episode; they work night shifts; they have an ongoing alcohol or substance abuse problem, or sleep apnea or other confounding sleep disorders, or medical conditions that may account for a sleep disturbance; or they have conditions or cognitive impairments incompatible with participation. Patients must not have been in a defined episode in the previous month or have been hospitalized in the previous two months. Any prophylactic medication of the participants must be accepted by a doctor in the study.

**Figure 1:**
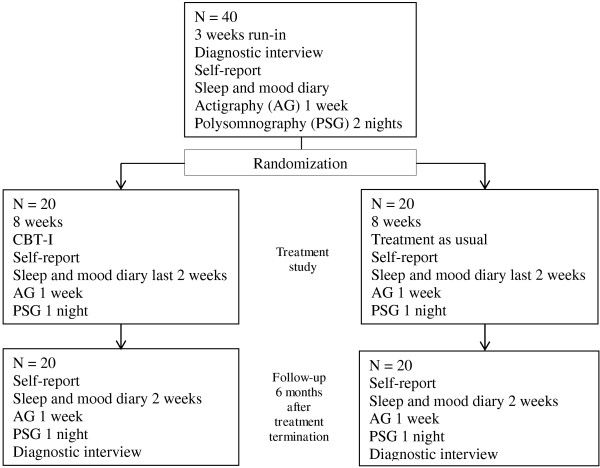
Study design.

Patients meeting criteria for inclusion will enter a run-in phase consisting of three weeks, during which they will complete a sleep and mood diary and a register of adherence to medication. In the last of these three weeks, they will be monitored for seven consecutive days with an actigraph and for two nights using ambulatory polysomnography. The patients will be assigned a patient number and sign a consent form after receiving oral and written information about the study prior to undergoing study procedures. The patients will receive US $85 in compensation for each polysomnography session, and another US $85 in compensation for transport costs and other expenses related to participation. Those patients who are able to complete the three-week run-in phase and meet diagnostic criteria for insomnia will then be randomized to the treatment trial. Figure 1 summarizes the study design and Table 1 summarizes the measures to be used in the trial. Patients who have not received psychoeducation for bipolar disorder, either in groups or individually, will be offered such psychoeducation, to ensure that they have received optimal treatment for bipolar disorder. The patients will be rediagnosed for insomnia 6 months after treatment termination. The results of the nights recorded with sleep diaries, actigraphs, and polysomnography will be compared in order to describe the accuracy and clinical utility of sleep diaries and actigraphy compared with polysomnography in bipolar disorder. Participants in the TAU group will be offered standard treatment at the sleep clinic for their sleep disturbances at the end of the six-month follow-up. The included bipolar disorder patients will also be included in a descriptive study of bipolar patients in Norway, the Bipolar Research and Innovation Network- Norway (BRAIN-Norway).

**Table 1:** Measures in the treatment trial

	**Inclusion**	**Treatment**	**8 weeks**	**6 months**
*Clinical interviews*				
Structured Clinical Interview for DSM Disorders (SCID)	X			
Network Entry Questionnaire for Bipolar Disorder (NEQ)	X			
Montgomery Åsberg Depression Rating Scale (MADRS)	X			
Young Mania Rating Scale (YMRS)	X			
Insomnia Interview Schedule (IIS)	X			X
Insomnia criteria	X		X	X
*Registrations*				
Changes in medication	X	X	X	X
Hospital admissions	X	X	X	X
Consent	X			
*Objective measures*				
Actigraphy	X		X	X
Polysomnography	X		X	X
*Patient-reported measures*				
Sleep and mood diary	X		X	X
Beck Depression Inventory (BDI)	X		X	X
Beck Anxiety Inventory (BAI)	X		X	X
Altman Mania	X		X	X
Insomnia Interview Schedule (ISI)	X		X	X
Short-Form Health Survey (SF-36)	X		X	X
Flinders Fatigue Scale (FFS)	X		X	X
Morningness-Eveningness Questionnaire (MEQ)	X		X	X
Dysfunctional Attitudes and Beliefs about Sleep (DBAS-16)	X		X	X
Sleep-Related Behaviors Questionnaire (SRBQ)	X		X	X
Sleep Hygiene Index (SHI)	X		X	X
Insomnia Daytime Worry Scale (IDWS)	X		X	X

### Measures

#### Measures of affective disturbance

##### *Network Entry Questionnaire for Bipolar Disorder (NEQ)*

This is a Norwegian adaptation of NEQ used in the Bipolar Collaborative Network and in the Bipolar Research and Innovation Network, Norway. The NEQ gives a clinical description of the bipolar disorder, comorbidity, treatment history, and sociodemographics [15,16].

##### *Montgomery Åsberg Depression Rating Scale (MADRS)*

This is an interview-based questionnaire used to assess the level of depression [17]. Setting the cut-off value to 13 at baseline, instead of 9, takes into account that most participants in this study will probably score up to four points on the question regarding sleep.

##### *Young Mania Rating Scale (YMRS)*

This is an interview-based questionnaire used to assess level of mania or hypomania [18]. Setting the cut-off value to 7, instead of 6, which is normally used, takes into account that most participants in this study will probably score two points on the question regarding sleep.

##### *Beck Depression Inventory (BDI)*

This is a self-report questionnaire used to assess the level of depression [19]. It will be used before and after treatment, and at follow-up.

##### *Beck Anxiety Inventory (BAI)*

This is a self-report questionnaire used to assess the level of anxiety [20]. It will be used before and after treatment, and at follow-up.

##### *Altman Self-Rating Mania Scale*

This is a self-report questionnaire used to assess the amount of mania symptoms [21]. It will be used before and after treatment, and at follow-up.

*Dated registration* of changes in medication, hospitalizations, and other changes in care will be recorded from the start, after treatment (eighth week) and at the six-month follow-up.

### Measures of sleep disturbance and daytime impairment

We will use the recommendations for a standard research assessment of insomnia published by Buysse *et al*. [22].

#### *Polysomnography*

Sleep will be recorded with standard Somnoscreen equipment (EEG, EMG (submental and tibial, EOG, airflow, respiration, position and oxygen saturation), analyzed with Domino software. This will be done on two nonconsecutive nights during the last week of the run-in phase, one night post-treatment (eighth week) and one night at the six-month follow-up.

#### *Actigraphy*

Movement during sleep will be measured and stored continuously using a small wristwatch-like device. Actigraphs from Philips Respironics will be used and analyzed with Actiwatch Spectrum and the latest version of Actiware software. This will be done for one week at the end of the run-in phase, one week post-treatment (eighth week) and one week at the six-month follow-up. The medium sensitivity setting will be used, with an epoch length of 30 s.

#### *Sleep diary*

This will be kept daily during the three weeks before randomization, daily for two weeks at treatment termination and daily for two weeks at the six-month follow-up [14]. The sleep diary will include a rating of mood. The sleep diary will be kept in accordance with the consensus recommendation for sleep diaries [23].

#### *Insomnia Severity Index (ISI)*

This is a self-report questionnaire used to assess the level of insomnia severity [24]. Patients will complete the ISI before and after treatment and at the six-month follow-up. The ISI is the primary outcome measure.

#### *The Short-Form Health Survey (SF-36)*

This is a self-report questionnaire used to assess quality of life [25]. It will be used before and after treatment, and at follow-up.

#### *Flinders Fatigue Scale (FFS)*

This is a self-report questionnaire used to assess the degree of fatigue [26]. It will be used before and after treatment, and at follow-up.

#### *Morningness-Eveningness Questionnaire (MEQ)*[27]

This is a self-report questionnaire used to assess diurnal preference and circadian disturbance. It will be used before and after treatment, and at follow-up.

#### *Dysfunctional Attitudes and Beliefs about Sleep (DBAS-16)*[28]

This is a self-report questionnaire used to assess the degree to which patients hold dysfunctional beliefs about sleep. It will be used before and after treatment, and at follow-up.

#### *Sleep-Related Behaviors Questionnaire (SRBQ)*[29]

This is a self-report questionnaire used to assess the degree to which patients engage in safety behaviors to cope with poor sleep. It will be used before and after treatment, and at follow-up.

#### *Insomnia Daytime Worry Scale (IDWS)*[30]

This is a self-report questionnaire used to assess the amount of time patients spend worrying about sleep-related topics during the day. It will be used before and after treatment, and at follow-up.

#### *Sleep Hygiene Index (SHI)*

This is a self-report questionnaire used to determine how often the patient engages in behaviors that are considered to be incompatible with good sleep hygiene [31]. It will be used before and after treatment, and at follow-up.

### Data collection and randomization

Clinical interviews and registrations will be conducted by a psychiatrist, medical doctor, or clinical psychologist at the Bipolar Clinic, Østmarka Department of Psychiatry, St. Olav’s University Hospital, Trondheim, Norway. The insomnia diagnosis will be assessed before randomization, and at a six-month follow-up. The polysomnographs will be administered by the Department of Clinical Neurophysiology at St. Olav’s University Hospital and assessed by a blinded medical doctor. The actigraphy recordings will be administered by the clinician in charge of treatment of each patient. Except for the daily assessments of mood and sleep, the patient-reported measures will be collected using a secure internet-based interface, but patients will be given the option of completing the paper-version of the self-reported measures at the clinic instead.

Patients will be randomized to CBT-I or TAU by a web-based system developed and administered by the Unit of Applied Clinical Research, Norwegian University of Science and Technology (NTNU), Trondheim, Norway. Eligible patients who have signed the consent form and fulfill criteria for participation will be randomized sequentially, in blocks of variable size to ensure an even allocation throughout the study.

### Treatments

#### *Treatment as usual (TAU)*

This is pharmacological and supportive psychosocial treatment according to the needs and current regime of the patient. The amount of contact with health care professionals outside of our department will be registered. In addition, patients will be offered biweekly consultations with either a psychologist or a medical doctor from our study.

#### *Cognitive behavioral therapy for insomnia (CBT-I)*[14]

This is a multicomponent treatment usually consisting of one or more of the following interventions: sleep restriction therapy, psychoeducation about sleep, stimulus control, and challenging beliefs and perception of sleep. Bipolar patients may be prone to misperceive sleep for wakefulness [3,32]. There will also be a focus on challenging and correcting sleep state misperception using cognitive and behavioral techniques [33]. Circadian rhythm is likely to be disturbed in the included patients, so there will be particular emphasis on stabilization of the circadian rhythm. Chronotherapeutic interventions of light therapy and gradual sleep phase advancement are currently the best available treatment options for circadian rhythm disturbances [34]. If necessary, these interventions will be included, to stabilize the circadian rhythm. The treatment will last for three to eight sessions. The therapists will be two clinical psychologists and one psychiatrist. Therapists will have received training in CBT-I and have one to nine years of clinical experience with CBT-I. Patients will be given checks for treatment credibility. Therapy sessions will be checked for adherence to manual by having at least two therapists present during treatment.

### Withdrawal criteria

Patients will be withdrawn from the study if the clinician finds that the patient is in need of, or better served with, other treatment; if exclusion criteria are met; if the clinical condition gets significantly worse according to the clinicians’ judgment; or if patients withdraw their consent. All patients prematurely discontinuing the trial will be seen for a final evaluation. The date and reason for discontinuation will be noted.

### Sample size calculation

#### A-priori power calculations

The primary response variable, change in ISI score between baseline and endpoint, will be analyzed with the independent samples *t* test at a significance level of *α* = 0.05. Prior studies of CBT-I for patients with mental disorders and comorbid insomnia have found between-group effect sizes in a range from *d =*1.1 to *d =* 1.6 on the ISI. Thirty-six patients are required to have a 90% chance of detecting a between-group effect size of *d* = 1.1 at a significance level of *α =* 0.05*.* This corresponds to a change in ISI score from 18 in the control group to 14 in the CBT-I group given a standard deviation of 3.6.

In addition, secondary response variables of total sleep time, sleep efficiency, and changes in polysomnography parameters will be recorded. Daily and weekly variations in mood and time to relapse to a new episode will also be compared between the two groups.

### Statistics

Categorical variables will be analyzed using chi-square tests. Ordinal variables will be analyzed using nonparametric tests in addition to using Student’s *t* tests, analysis of variance (ANOVA) and analysis of covariance (ANCOVA) with baseline values as covariates. The time to change in treatment for bipolar disorder will be analyzed using survival analysis. Linear mixed models will be applied in analyses of treatment response. All patients who fulfill baseline registration will be included in the analysis of baseline characteristics. All subjects who receive at least one session of CBT-I and provide post-baseline efficacy measurements will be included in efficacy data analyses. Analyses including only patients fulfilling the treatment will also be conducted.

## Ethical approval

The study protocol has been approved by the Regional Committee for Medical Research Ethics in Middle Norway. Written informed consent will be obtained from all included patients.

## Discussion

This study will test whether treatment with CBT-I can provide improvement in sleep quality and will also be one of the first to test the effects of CBT-I on patients with insomnia and bipolar disorder. Insomnia is associated with several negative health outcomes in the general population [7]. It is likely that patients with bipolar disorder and insomnia, in addition to the same negative consequences, have an increased risk of experiencing new episodes of depression or (hypo) mania.

We are not aware of side effects related to CBT-I, but as it is an untested treatment in bipolar disorder, we do not know if it may trigger new episodes of depression or mania [35]. The extensive study program may become a potential problem for inclusion of participants and completion of study procedures. Motivational work will be all-important before and during treatment in both groups.

Descriptions of quality of sleep in euthymic periods of bipolar disorder are important for planning interventions and improving monitoring for signs of recurrence of new episodes of depression or (hypo) mania.

It is important to compare the value of diagnostic tools, such as sleep diaries, actigraphs and polysomnography, in order to improve the quality of diagnosis and possibly to simplify the clinical practice of such tools [36].

### Trial status

Inclusion of patients will start in August 2013 and is expected to continue until January 2015 or until the necessary number of participants has been included.

## Abbreviations

ANCOVA: analysis of covariance; ANOVA: analysis of variance; BAI: Beck Anxiety Inventory; CBT-I: cognitive behavioral therapy for insomnia; BDI: Beck Depression Inventory; DBAS: Dysfunctional Attitudes and Beliefs about Sleep; FFS: Flinders Fatigue Scale; IDWS: Insomnia Daytime Worry Scale; IIS: Insomnia Interview Schedule; ISI: Insomnia Severity Index; MADRS: Montgomery Åsberg Depression Rating Scale; MEQ: Morningness-Eveningness Questionnaire; NEQ: Network Entry Questionnaire for Bipolar Disorder; SCID: Structured Clinical Interview for DSM Disorders; SF-36: Short-Form Health Survey; SHI: Sleep Hygiene Index; SRBQ: Sleep-Related Behaviors Questionnaire; TAU: treatment as usual; YMRS: Young Mania Rating Scale.

## Competing interests

The authors declare that they have no competing interests.

## Authors’ contributions

MKS, KKG, KL, TS, HK, and GM all conceptualized the study, participated in the study design and contributed to the writing of the study protocol, drafting and editing of this manuscript. GM is the overall study principal investigator. All authors read and approved the final manuscript.
